# The Multifaceted Nature of Streptococcal Antigen I/II Proteins in Colonization and Disease Pathogenesis

**DOI:** 10.3389/fmicb.2020.602305

**Published:** 2020-11-25

**Authors:** Haider S. Manzer, Angela H. Nobbs, Kelly S. Doran

**Affiliations:** ^1^Department of Immunology and Microbiology, University of Colorado Anschutz Medical Campus, Aurora, CO, United States; ^2^Bristol Dental School, University of Bristol, Bristol, United Kingdom

**Keywords:** antigen I/II, *Streptococcus*, adhesin, dental caries, respiratory infection, vaginal colonization, meningitis, biofilms

## Abstract

Streptococci are Gram-positive bacteria that belong to the natural microbiota of humans and animals. Certain streptococcal species are known as opportunistic pathogens with the potential to cause severe invasive disease. Antigen I/II (AgI/II) family proteins are sortase anchored cell surface adhesins that are nearly ubiquitous across streptococci and contribute to many streptococcal diseases, including dental caries, respiratory tract infections, and meningitis. They appear to be multifunctional adhesins with affinities to various host substrata, acting to mediate attachment to host surfaces and stimulate immune responses from the colonized host. Here we will review the literature including recent work that has demonstrated the multifaceted nature of AgI/II family proteins, focusing on their overlapping and distinct functions and their important contribution to streptococcal colonization and disease.

## Introduction

The genus *Streptococcus* is a heterogeneous group of Gram-positive bacteria that can be part of the natural microbiota. Various streptococci are commonly isolated from the oral cavity, intestines, or female reproductive tracts in healthy humans. These colonizing streptococci are often commensal organisms that persist without causing disease. In some cases, colonization by commensal streptococci can actually benefit the host through mechanisms such as niche competition or direct inhibition of more pathogenic organisms (Abranches et al., 2018; Nobbs and Kreth, 2019); however, there are also many streptococcal species that are opportunistic pathogens with the potential to cause severe invasive disease. Within the viridans group of streptococci, a heterogeneous group consisting of typically commensal α-hemolytic species, exists *Streptococcus mutans*. *S. mutans* is a major agent of dental caries (tooth decay) (Lehner et al., 1975) and infective endocarditis (Hoen et al., 2002). *Streptococcus pyogenes* (Group A *Streptococcus*, GAS) is a major cause of skin and soft tissue infections, as well as respiratory infections (Steer et al., 2012). Similarly, *Streptococcus agalactiae* (Group B *Streptococcus*, GBS) is known to colonize the female reproductive tract and is a leading cause of neonatal sepsis, pneumonia, and meningitis (Edmond et al., 2012). Understanding the shared mechanisms by which these diverse species colonize and cause disease is the first step toward treatment and prevention. One example of a shared virulence factor among these species is the family of multifunctional streptococcal surface anchored adhesins known as antigen I/II (AgI/II) proteins.

Mike Russell et al. and Roy Russell et al. identified the cell wall antigens I and II in *Streptococcus mutans* nearly simultaneously over 40 years ago (Russell and Lehner, 1978; Russell, 1979; Russell et al., 1980). Soon after, it was shown that antigen II was actually a breakdown product of antigen I (Kelly et al., 1989), giving rise to their new classification as antigen I/II proteins. These AgI/II proteins are widely distributed not only among various serotypes within *S. mutans* (Russell and Lehner, 1978; Russell et al., 1980; Ma et al., 1991), but orthologous proteins sharing similar structure and functions also exist within a number of other streptococcal species (Kelly et al., 1989; Jenkinson and Demuth, 1997; Brady et al., 2010). Due in part to the initial discovery of AgI/II proteins within *S. mutans*, their contribution to dental caries has been a major focus of AgI/II related research; however, more recent studies have investigated AgI/II proteins in other species and biological niches as well. The multifunctional nature of these adhesins in multiple niches may directly contribute to the transition toward pathogenesis as the same protein that promotes colonization of a commensal or beneficial organism in one niche may also allow persistence after that organism gains access to new and potentially more susceptible host tissues. Furthermore, discrete locations of various binding sites on AgI/II proteins for interaction with different microorganisms and host molecules ensures maximum protein functionality, which could be a key mechanism for generating diversity in the development of microbial communities. In addition to the cariogenic properties of AgI/II proteins, these studies have revealed diverse functions ranging from adherence to immune modulation, which in turn contribute to streptococcal colonization and disease.

## Nomenclature

Proteins belonging to the AgI/II family that have been identified to date are known by various names, many of which are shown in Table 1. The majority of these were independently studied and named by different groups prior to the widespread availability of sequencing technologies that eventually revealed that these were, in fact, the same proteins. For example, the single *S. mutans* AgI/II protein is known by at least seven different names. In some cases, the different names represent different proteins coded by unique genes within the same species; BspA-D in GBS, SspA/B in *S. gordonii*, and PAaA/B in *S. criceti* are examples of this. Standardization of the nomenclature in future publications would significantly clarify which proteins are being discussed. A few groups have used the “AgI/II” designation for antigen I/II proteins regardless of the species to which the protein belongs (Sciotti et al., 1997; Chuzeville et al., 2017; Auger et al., 2019).

**TABLE 1:** AgI/II family protein names by species.

Species	Protein name(s)	Reference(s)
*S. mutans*	SpaP/P1/PAc/Sr/MSL-1/AgB/IF	Russell and Lehner, 1978; Russell et al., 1980; Kelly et al., 1989; Demuth et al., 1990b; Ogier et al., 1990; Bleiweis et al., 1992; Jenkinson and Demuth, 1997
*S. gordonii*	SspA/SspB	Demuth et al., 1996; Brooks et al., 1997; Jenkinson and Demuth, 1997
*S. agalactiae*	BspA-D/PAc/SSP-5	Tettelin et al., 2005; Rego et al., 2016
*S. pyogenes*	Spy1352/AspA	Zhang et al., 2006
*S. sobrinus*	SpaA/PAg	LaPolla et al., 1991
*S. sanguinis*	SSP-5/PAc	Demuth et al., 1988, 1990a, b
*S. intermedius*	Pas	Petersen et al., 2001, 2002; Tamura et al., 2001
*S. criceti*	PAaA/PAaB	Tamura et al., 2001, 2004
*S. downei*	PAh	Tamura et al., 2008
*S. suis*	SSP-5/AgI/II	Chuzeville et al., 2017; Auger et al., 2019

*The most common names used to refer to AgI/II proteins from different Streptococcus species are listed.*

## Prevalence and Conservation

**FIGURE 1:**
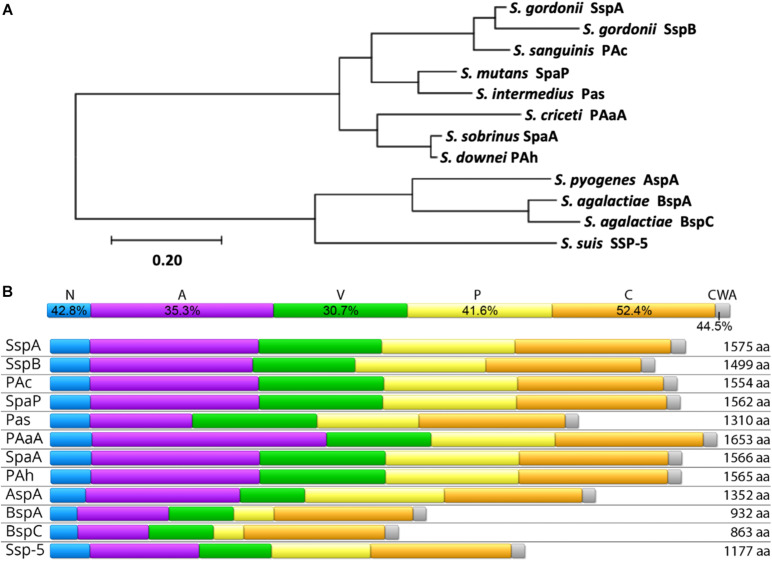
Homology of AgI/II Family Proteins. **(A)** The evolutionary history was inferred by using the Maximum Likelihood method and JTT matrix-based model (Jones et al., 1992) using full-length amino acid sequences of the indicated proteins. The tree with the highest log likelihood (–21701.96) is shown. The tree is drawn to scale, with branch lengths measured in the number of substitutions per site. Evolutionary analyses were conducted in MEGA X (Kumar et al., 2018). **(B)** Conservation of individual domains is shown (top) as the pairwise identity percentage resulting from a MUSCLE protein alignment of each individual domain, as well as the primary structure and amino acid length of AgI/II proteins from select streptococci (bottom). The domains shown are the N-terminal region (N; blue), alanine-rich repeats (A; purple), the variable region (V; green), proline-rich repeats (P; yellow), the C-terminal region (C; orange), and the cell wall anchoring region (CWA; gray). The Uniprot IDs for protein sequences used in both **(A,B)** are as follows: *S. mutans* SpaP (P23504), *S. gordonii* SspA (Q54185), *S. gordonii* SspB (Q54186), *S. agalactiae* BspA (Q8E589), *S. agalactiae* BspC (A0A380IJX7), *S. pyogenes* AspA (Q48S75), *S. intermedius* Pas (Q9KW51), *S. sobrinus* SpaA (Q53414), *S. sanguinis* PAc (F3V086), *S. criceti* PAaA (Q9LBG3), *S. downei* PAh (Q59HN9), and *S. suis* SSP-5 (A0A0Z8CV71).

AgI/II proteins are nearly ubiquitous within streptococci. In order to examine the relatedness of AgI/II proteins across species, representative genes from those listed in Table 1 were aligned, and their phylogenetic relationships are shown in Figure 1A. Orthologous genes, many of which have yet to be further characterized and may not be expressed at the protein level, were also identified in *S. rattus, S. oralis, S. mitis, S. anginosus, S. constellatus, S. vestibularis*, and *S. parasanguinis* via amplification from *S. mutans* SpaP based PCR probes (Ma et al., 1991). Another potential AgI/II protein was recently identified in *S. salivarius* as well, though it also remains to be characterized (Chaffanel et al., 2018). As AgI/II genes are so widely distributed among streptococci, it is likely that other species that were not listed here also contain AgI/II orthologs that have yet to be identified. For example, while there was previously no evidence for an AgI/II gene within *S. pneumoniae* (Brady et al., 2010), a BLASTP search for homologs of the GAS AgI/II gene AspA within *S. pneumoniae* strains revealed a homologous protein in a newly sequenced strain (NCBI RefSeq: WP_160544519.1). This protein has a similar primary structure with 69% pairwise identity to AspA, with 83% coverage, which is comparable to the variation that exists between AgI/II genes from different species. While this was the only *S. pneumoniae* strain we found containing an AgI/II homolog, its presence nonetheless highlights the widespread distribution of AgI/II proteins and their potential to provide pathogens with a selective advantage in certain niches.

Previous studies comparing sequences across AgI/II genes from multiple species have revealed domain-based variation in conservation (Ma et al., 1991; Brady et al., 2010; Deng et al., 2019). This is shown to be especially true when protein sequences from a larger number of species are compared (Figure 1B). Comparisons of AgI/II genes from multiple strains within one species, however, reveal high levels of sequence conservation (Brady et al., 1991; Ma et al., 1991; Do et al., 2010; Chuzeville et al., 2015; Rego et al., 2016). For example, a study comparing 37 *S. mutans* SpaP genes observed non-synonymous polymorphisms in only 4% of sites (Do et al., 2010). This indicates both diversity within the AgI/II family of proteins, as well as specificity, where the AgI/II proteins within each species may have evolved to potentially better interact with niche specific receptors. The fact that AgI/II genes from more invasive streptococcal species cluster separately from the viridans streptococci supports this theory (Figure 1A). One factor contributing to the distribution and clustering of AgI/II genes is their ability to be shared via horizontal gene transfer. An example of this is seen with the GAS and GBS AgI/II genes. In GAS, the AgI/II genes are located on the integrative and conjugative element named region of difference 2 (RD2), which is thought to be horizontally shared between GBS, conferring pathogenic traits that support invasive disease in pregnant women and neonates (Zhang et al., 2006; Sitkiewicz et al., 2011; Chuzeville et al., 2015; Deng et al., 2019; Jain et al., 2019). It is especially concerning that this virulence factor is able to spread horizontally since this family of proteins has a wide variety of pathogenic functions, and their presence in streptococcal species occupying unique niches may correlate with the ability to cause disease as well.

## Structure

**FIGURE 2:**
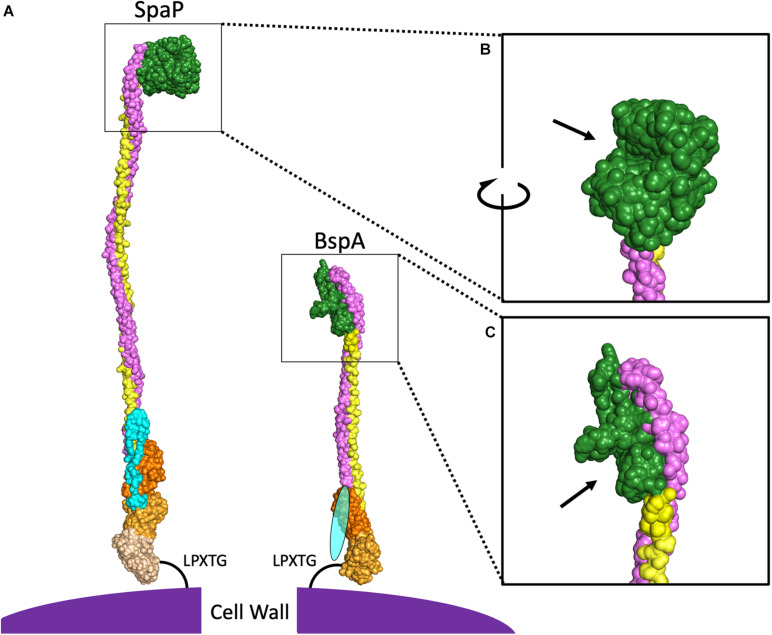
Comparison of SpaP and BspA Protein Structures. **(A)** PyMOL was used to combine the published models for domains of SpaP (N-terminus PDB:4TSH, A3VP1 PDB:3IOX, C-terminus PDB:6E3F) and BspA (V-domain, C-terminus PDB:5DZA). Additional SpaP A and P repeats were modeled from A3VP1, as were the A- and P-domains for BspA. As there is no structure for the N-terminal domain of any AgI/II protein among the cluster of invasive species shown in Figure 1A, the BspA N-terminus is depicted by an oval. Light blue indicates the N-terminal domain, purple indicates the A-domain, green indicates the V-domain, yellow indicates the P-domain, and the shades of orange indicate different globular domains at the C-terminus. The V-domain of SpaP **(B)** and BspA **(C)** have been enlarged with arrows pointing to binding pockets.

The primary structure of AgI/II proteins is shown in Figure 1B. AgI/II proteins begin with an N-terminal domain containing a signal peptide mediating secretion via the general secretory pathway (Scott and Barnett, 2006). This is adjacent to the A-domain, a region containing multiple alanine-rich repeats (Kelly et al., 1989). The A-domain is connected to a globular domain termed the V-domain based on the variability observed in this region between strains (Brady et al., 1991). The V-domain contains a putative binding pocket, which will be elaborated on in greater detail further on. At the other end of the V-domain is the P-domain, termed for the presence of proline-rich repeats within this region (Kelly et al., 1995). Following the P-domain is the C-terminal region, which consists of two or three globular IgG-like domains. Finally, there is an LPXTG motif for sortase-mediated cell wall anchoring (Kelly et al., 1989). Overall, the AgI/II proteins adopt a cell wall-anchored stalk structure formed by interactions between the intertwined A- and P-domains, which lifts the globular V-domain over 50 nm away from the cell surface in the case of the *S. mutans* SpaP (Larson et al., 2010). The A- and P-domains of SpaP were shown to utilize a unique interlocking between tyrosine residues from heptad repeat motifs in the A-domain with hydrophobic pockets consisting of PxxP motifs within the P-region (Larson et al., 2010). The A- and P-domains are significantly smaller in some homologs such as the Bsp proteins of GBS (Figures 1B, 2), and it has also been shown that the interaction between A- and P-domains within these homologs is formed in a different manner. The A-domain of BspA contains an asparagine seam whose hydrogens interact with oxygen and nitrogen atoms within the P-domain (Rego et al., 2016). In addition to the A- and P-domains interacting, the tertiary stalk structure of AgI/II proteins is further stabilized by interactions between the N- and C-terminal domains in SpaP (Heim et al., 2014). As the structure of the N-terminal domain in other homologous proteins has not been experimentally determined thus far, the level of conservation of such a locking mechanism remains to be seen.

In addition to projecting the V-domain away from the cell surface to improve interactions with ligands, this stable stalk structure allows for a degree of flexibility that is known to promote bacterial attachment under shear conditions. While this tertiary structure is highly conserved, there is regional variability particular to individual species, especially at the V-domain. Interestingly, the full-length protein sequences used to generate the phylogenetic tree shown in Figure 1A cluster into a group of viridans streptococci and a separate group of more traditionally invasive species. Representatives of these two AgI/II protein structures, SpaP from *S. mutans* and BspA from GBS are shown in Figure 2. These two species were chosen because each has the largest number of domains with experimentally determined structures published within each cluster. The structural differences between homologs contribute to both overlapping and distinct functions for AgI/II proteins within streptococci. For example, AgI/II proteins from some species are able to bind to sialic acids, while those from other species cannot (Demuth et al., 1990a, b; Soell et al., 1994; Vernier et al., 1996; Chatenay-Rivauday et al., 1998). Structural differences exist even within the phylogenetic clusters, as seen by variations in the published V-domain structure from *S. gordonii* SspB (Forsgren et al., 2009) from that of SpaP. These types of species-dependent variations in AgI/II function will be discussed in greater detail in the following sections. It is likely that some of these functional variations also depend on structural differences in domains other than the V-domain. While the full structure of SpaP has been mostly solved (Heim et al., 2014, 2015), other than the C-domain of *S. pyogenes* AspA (Hall et al., 2014) and GBS BspA V- and C-domains (Rego et al., 2016), the structures of additional AgI/II domains from the cluster of invasive streptococci have not been experimentally determined. A better understanding of the structural differences between these AgI/II orthologs may reveal key insights regarding their unique mechanisms for colonization or disease progression. Additionally, the structure of *Enterococcus faecalis* Asc10 displays a primary structure akin to that of AgI/II proteins (Waters et al., 2004; Chuang et al., 2009). The region analogous to the AgI/II V-domain of Asc10 was recently shown to have a similar structure to the V-domain of *S. gordonii* SspB (Schmitt et al., 2018). As such, gaining a better understanding of the AgI/II structure may indirectly provide information regarding the mechanisms for pathogenesis utilized by other microorganisms as well.

## Adhesive Function

### Functional Amyloids and Auto-Aggregation

One well known function of AgI/II proteins is their direct contribution to auto-aggregation of streptococci without intermediate molecules (Demuth et al., 1990a; Jenkinson and Demuth, 1997; Brady et al., 2010; Maddocks et al., 2011; Chuzeville et al., 2017; Deng et al., 2019). In recent years, it has also been shown that fragments of *S. mutans* SpaP interact with each other to form functional amyloid fibers (Oli et al., 2012). This discovery prompted investigations regarding the molecular interactions contributing to this phenomenon. As mentioned earlier, AgI/II proteins are known to fragment, releasing what was previously known as antigen II. These released fragments can interact with surface anchored AgI/II proteins, particularly through the V-domain of the surface anchored protein with either the V- or C-domains of the fragmented protein (Heim et al., 2015; Rego et al., 2016; Tang et al., 2016; Rivière et al., 2020). AgI/II fragments comprised of the C-domain are able to form the functional amyloid fibers *in vitro* (Tang et al., 2016; Barran-Berdon et al., 2020). Furthermore, AgI/II functional amyloids contribute to stabilization of *S. mutans* biofilms (Oli et al., 2012; Tang et al., 2016; Barran-Berdon et al., 2020). Together, this information suggests a critical role of AgI/II protein interactions for the stabilization of streptococcal communities, as well as in the formation of a structural foundation in biofilms that may be taken advantage of by other species. The direct role of AgI/II proteins in polymicrobial biofilms at various tissues will be discussed below.

### Extracellular Matrix Interactions

In addition to auto-interactions, AgI/II proteins are able to interact with a large number of host ligands. As a class of multifunction adhesins, AgI/II proteins play a role in streptococcal interactions with extracellular matrix (ECM), or canonical ECM proteins outside of the context of ECM, as seen with some components of the salivary pellicle. These interactions potentially facilitate adherence to both mucosal surfaces such as the lung or vaginal epithelium, as well as non-mucosal surfaces such as endothelial cells or teeth. Thus far, interactions with the fibrous components of the ECM has been a major focus of research, especially collagen, fibrinogen, fibronectin, and laminin. As collagen is a major component of dentin (Linde, 1989), there has been a large interest in collagen-binding proteins and their role in mediating streptococcal access and invasion into tooth surfaces. It has been shown that *S. gordonii* SspA and SspB (Love et al., 1997; Heddle et al., 2003), *S. suis* AgI/II (Chuzeville et al., 2017), and *S. mutans* SpaP (Love et al., 1997; Sciotti et al., 1997; Beg et al., 2002; Petersen et al., 2002; Sullan et al., 2015; Esberg et al., 2017) interact with and promote adherence to surfaces coated with type I collagen, while *S. intermedius* Pas does not (Petersen et al., 2001, 2002). Some of these studies utilized human collagen (Sciotti et al., 1997; Beg et al., 2002; Chuzeville et al., 2017), while others used rat tail collagen (Love et al., 1997; Sullan et al., 2015). This is significant because a study that used type I collagen from both human and rat tail found that *S. mutans* SpaP interacts with rat and not human collagen (Petersen et al., 2002). Differences in experimental set up might explain these contradictory results, but additional investigation is required to definitively determine the role of an AgI/II – collagen interaction within the setting of human disease.

Streptococcal binding of laminin may also play a role in colonization or invasion of various tissues (Singh et al., 2012). Studies have shown that in addition to *S. intermedius* Pas (Petersen et al., 2001), *S. mutans* SpaP (Sciotti et al., 1997; Busscher et al., 2007) also interacts with laminin. Another study saw a minor decrease in the ability of a *S. mutans* SpaP knockout to bind human laminin but as the difference was not significant, the authors concluded that SpaP did not contribute to laminin binding (Beg et al., 2002); thus, additional investigations into the interactions of AgI/II proteins with laminin are still needed. Certain streptococcal species are also major agents of infective endocarditis (IE), and the ability to bind fibronectin and fibrinogen in addition to collagen may contribute to development of this disease (Baddour, 1994). *S. mutans* SpaP (Sciotti et al., 1997; Beg et al., 2002; Petersen et al., 2002; Kelemen et al., 2004; Sullan et al., 2015), *S. intermedius* Pas (Petersen et al., 2001, 2002), and *S. suis* AgI/II (Chuzeville et al., 2017) have all been shown to bind fibronectin. Additionally, *S. mutans* SpaP (Beg et al., 2002; Kelemen et al., 2004), *S. suis* AgI/II (Chuzeville et al., 2017), and GBS BspC (Chuzeville et al., 2015) have been shown to bind fibrinogen. The role of AgI/II proteins in binding of other ECM components such as elastin is still a significant knowledge gap that warrants future investigation.

### Additional Host Ligands

Direct AgI/II interactions with host cells are also well documented. These proteins mediate attachment to epithelial (Vernier et al., 1996; Sciotti et al., 1997; Chuzeville et al., 2017; Chaffanel et al., 2018; Pidwill et al., 2018; Jain et al., 2019) and endothelial (Vernier et al., 1996; Al-Okla et al., 1999; Deng et al., 2019; Oho and Nagata, 2019) cells, in addition to monocytes and fibroblasts (Soell et al., 1994; Chatenay-Rivauday et al., 1998, 2000; Petersen et al., 2001; Hajishengallis et al., 2002; Engels-Deutsch et al., 2003). The diversity of different cell types that streptococci adhere to using AgI/II proteins highlights their dynamic role as adhesins. One ligand for *S. mutans* SpaP that was identified early on is the intermediate filament (IF) protein, keratin (Sciotti et al., 1997). More recently, vimentin, a type III IF protein, was also identified as the GBS BspC receptor mediating adherence to human cerebral microvascular endothelial cells (hCMECs) (Deng et al., 2019). Despite the ubiquitous nature of IF proteins as structural components in all cell types, they remain severely understudied as receptors for AgI/II proteins, and future studies should investigate the role of potential IF-AgI/II interactions in pathogenesis.

Salivary agglutinin (SAG) is perhaps the best documented host receptor for AgI/II proteins and will be discussed in a dedicated section below; however, these investigations have revealed the lectin-like properties of AgI/II proteins. For example, sialic acid (and to a lesser degree, fructose, and mannose) binding by *S. sanguinis* SSP-5 competitively inhibits aggregation and interaction with SAG (Demuth et al., 1990a). Conversely, *S. mutans* SpaP interaction with SAG was inhibited by fucose and lactose, but not by sialic acid (Demuth et al., 1990b). Interestingly, *S. mutans* AgI/II was shown to bind *N*-acetylneuraminic acid (a sialic acid) in addition to fucose on the surface of monocytes and KB cells (Soell et al., 1994; Vernier et al., 1996; Chatenay-Rivauday et al., 1998). Additionally, the V-domain of AgI/II proteins is thought to contain a carbohydrate-binding pocket (Troffer-Charlier et al., 2002; Forsgren et al., 2009; Rego et al., 2016), and another cleft between two of the globular C-terminal domains of SpaP was shown to bind glucose (Larson et al., 2011). Collectively, this information indicates that while members of the AgI/II family of proteins have similar and often overlapping specificity for host factors, many of these interactions may be unique to both the AgI/II protein expressed in certain species, as well as the cell type in which the interaction is taking place.

AgI/II proteins are also able to interact with a number of receptors involved in immune signaling. *S. gordonii* SspA and SspB have been shown to interact with α5β1 integrins on human lung epithelial A549 cells, HEp-2 cells, and coated plates (Nobbs et al., 2007; Andrian et al., 2012). Furthermore, the interaction between SspA/SspB with α5β1 integrins was shown to be mediated via the N-terminal portion of the protein, and this interaction contributed to invasion into HEp-2 cells in addition to adherence (Nobbs et al., 2007). Due to the widespread expression of α5β1 integrins in multiple cell types, it is likely that their interaction with AgI/II proteins contributes to adherence and invasion in other cell types as well, but this has yet to be confirmed.

## Interactions With the Immune System

### Phagocytosis

AgI/II proteins are able to interact with or evade the host immune system, further impacting disease. One main niche where this occurs is within the bloodstream, as streptococci are often associated with blood-borne systemic diseases. AgI/II proteins have anti-phagocytic functions that may promote survival within the host and development of high levels of bacteremia. An *S. suis* AgI/II deficient mutant was more readily internalized by resident peritoneal macrophages but had no difference from wildtype in intracellular survival (Auger et al., 2019). The knockout strain also displayed increased internalization by dendritic cells, along with decreased intracellular survival within the dendritic cells. Furthermore, the *S. suis* AgI/II knockout was killed more readily when exposed to whole murine blood, collectively resulting in a dramatic difference in both rapid clearance of the knockout strain from murine blood and mouse survival upon intraperitoneal or intravenous infection (Auger et al., 2019). Similarly, deletion of AspA in GAS led to increased killing by the human neutrophil (HL60) and mouse macrophage (J774.2) cell lines, while expressing AspA in *L. lactis* led to decreased killing by the same cell lines (Franklin et al., 2013). Interestingly, heterologous expression of *S. gordonii* SspB within *L. lactis* had no impact on phagocytosis, indicating species specific differences in the sufficiency of AgI/II proteins to impact phagocytosis. This is also supported by the fact that the opposite trend was observed with *S. mutans* PAc, where a PAc knockout and *S. mutans* clinical strains with less AgI/II surface expression exhibited a decreased ability to be phagocytosed by human polymorphonuclear leukocytes (Nakano et al., 2006). Furthermore, the PAc deficient strain was not cleared as efficiently as wildtype *S. mutans* after intravenous injection within a rat model of bacteremia.

### Immune Signaling

AgI/II proteins have also been shown to modulate immune responses while in the bloodstream. In the same rat model for bacteremia that was mentioned above, significantly higher concentrations of serum sialic acid [a marker for systemic inflammation (Rajendiran et al., 2014)] were observed after injection with the PAc deficient mutant as compared to the wildtype control (Nakano et al., 2006). In this case, presence of the AgI/II protein appeared to dampen inflammation, although the opposite was observed in other *S. mutans* studies. As mentioned earlier, AgI/II proteins interact with THP-1 monocytes derived from human peripheral blood. *S. mutans* SpaP was able to induce release of the neutrophil chemoattractant IL-8 by THP-1 cells (Engels-Deutsch et al., 2003). SpaP also induced the pro-inflammatory cytokines TNFα and IL-1β from THP-1 cells, which was partially mediated by CD14 and TLR4, but not significantly by TLR2 (Hajishengallis et al., 2002). *S. mutans* Sr was similarly shown to bind human monocytes and induce the release of TNF, IL-1, and IL-6 (Soell et al., 1994). These seemingly contradictory results indicate complex interactions that strike a delicate balance with the host immune system. The mechanistic details regarding the contribution of *S. mutans* AgI/II to immune suppression or activation should be investigated in greater detail.

Despite the controversy involved with SpaP, most other AgI/II proteins appear to induce a pro-inflammatory response by cells found within the blood. *S. intermedius* Pas has been shown to induce IL-8 from THP-1 cells (Petersen et al., 2001). *S. gordonii* SspA and SspB both also induce TNF, IL-6, IL-10, and IL-12 release by bone marrow derived dendritic cells (Andrian et al., 2012). Intraperitoneal infection of mice with a *S. suis* AgI/II mutant strain led to decreased levels of a large number of pro-inflammatory mediators within the plasma, including IL-6, IL-12p70, IFN-γ, CCL2, CCL3, CCL4, CXCL1, and CXCL2 (Auger et al., 2019). Additionally, TLR2 and TLR4 were shown to contribute to MyD88 dependent induction of TNF, IL-1β, IL-6, and CCL3 in dendritic cells (Auger et al., 2019). TLR2 and TLR4 both interact with CD14 to sense extracellular factors and begin signal transduction (Arroyo-Espliguero et al., 2004). One study showed that THP-1 cells treated with vitamin D_3_ (known to increase expression of CD14) were not stimulated to a higher degree by *S. mutans* SpaP and concluded that CD14 was not a receptor for the protein (Chatenay-Rivauday et al., 1998); however, another study soon after showed that monoclonal antibodies specific to CD14 and TLR4 (but not TLR2), both decreased immune activation by SpaP in THP-1 cells (Hajishengallis et al., 2002). This indicates that SpaP does likely interact with CD14, but requires the intracellular signaling domain of TLR4 to induce the expected immune responses. Collectively, this information suggests an overwhelmingly pro-inflammatory response to AgI/II proteins by white blood cells. Still, as there is evidence for immune suppressive or anti-phagocytic characteristics of some of the AgI/II proteins, it remains possible that these AgI/II proteins have similarly complex interactions with the immune system under conditions or niches that have yet to be investigated.

AgI/II proteins also promote inflammatory signaling in other cell types, including epithelial and endothelial cells. As mentioned previously, AgI/II proteins can bind α5β1 integrins, which are known to sense the extracellular environment through adhesive interactions and initiate signaling cascades that alter a variety of host processes (Winograd-Katz et al., 2014). *S. mutans* SpaP is able to bind to α5β1 integrins on human saphenous vein endothelial cells (HSVECs) and osteoblasts, contributing to inflammatory signaling (Al-Okla et al., 1999; Son et al., 2012). AgI/II binding of α5β1 on the HSVEC surface was shown to induce the MAPK signaling pathway and tyrosine phosphorylation of phospholipase Cγ, focal adhesion kinase, and paxillin (Al-Okla et al., 1999). This eventually leads to IL-8 release in a PI3K independent manner. SpaP induction of HSVEC IL-8 release was confirmed by another study as well, along with release of IL-6 (Vernier et al., 1996). *S. gordonii* SspA and SspB also increased IL-6, MCP-1 and IL-8 production in A549 cells, and induction of both IL-6 and MCP-1 could be inhibited with antibodies that blocked β1 integrin. Furthermore, SspA and SspB induced IL-6 and IL-8 within HEp-2 cells, as well as CXCL1 and CXCL2 in lung homogenates (Andrian et al., 2012). Similarly, the GBS AgI/II protein known as BspC was shown to induce inflammatory signaling in hCMECs leading to meningitis-associated inflammation. BspC induced IL-8, IL-1β, and CXCL1 release by hCMECs, and also KC protein and IL-1β within a murine model of hematogenous meningitis (Deng et al., 2019). This is a clear case where the AgI/II mediated inflammatory signaling contributes to disease. Given the large body of data showing that similar immune induction occurs at other body sites, the direct role of AgI/II proteins in inflammatory disease should be investigated in greater detail.

## Niche Specific Interactions

### Oral Cavity

As mentioned previously, due to the initial discovery of AgI/II proteins in *S. mutans*, the AgI/II proteins of streptococci that colonize the oral cavity are the most well studied. Within the oral cavity, AgI/II proteins are known to interact with one of the host innate defense proteins known as salivary agglutinin (SAG). Initial studies identified that *S. mutans* SpaP (Brady et al., 1992; Hajishengallis et al., 1994; Sciotti et al., 1997; Ahn et al., 2008), *S. sanguinis* PAc (Demuth et al., 1988, 1990a; Lamont et al., 1991), *S. sobrinus* SpaA (Lamont et al., 1991), and *S. gordonii* SspA (Jenkinson et al., 1993) all interacted with fluid-phase SAG, resulting in aggregation. The aggregation acts as a defense mechanism in this case as it promotes clearance of bacteria through actions such as swallowing. It has since been discovered that SAG and the lung scavenger receptor protein known as glycoprotein-340 (gp340) [also known as Deleted in Malignant Brain Tumors 1 or DMBT1 (Mollenhauer et al., 1997)] are the same protein (Prakobphol et al., 2000). In the context of mucosal colonization, the protein has historically been referred to as either SAG or more recently gp340, so it will be simply referred to as SAG/gp340 in this review. Within the oral cavity, SAG/gp340 can exist in fluid phase as a salivary component, or in an adsorbed phase that can mediate bacterial attachment to the tooth surface, thereby contributing to dental plaque biofilm formation (Madsen et al., 2010). Interestingly, it seems that AgI/II proteins have varying interactions with these two phases of SAG/gp340. *S. gordonii* SspA and SspB, *S. intermedius* Pas and *S. mutans* SpaP all contributed to aggregation by fluid-phase SAG/gp340 at comparable levels, but only the AgI/II proteins of *S. gordonii* mediated higher affinity binding to surface-bound SAG/gp340 (Loimaranta et al., 2005; Maddocks et al., 2011). Likewise, while GAS AspA contributes to attachment to immobilized SAG/gp340, it did not cause aggregation by fluid-phase SAG/gp340 (Maddocks et al., 2011). The AgI/II protein of *S. suis* has also been shown to interact with both fluid-phase and immobilized SAG/gp340, contributing to both aggregation and surface adherence, respectively (Chuzeville et al., 2017). While GAS and *S. suis* are not common colonizers of the oral cavity, the ability for their AgI/II proteins to interact with SAG/gp340 highlights the conservation of this particular function.

An early study using monoclonal blocking antibodies revealed that binding to fluid-phase and surface-immobilized SAG/gp340 is mediated by different regions of the AgI/II protein (Brady et al., 1992). This was the first indication that different portions of SAG/gp340 are accessible to AgI/II binding in its two physical states. Multiple studies have investigated this phenomenon in more detail, specifically examining the scavenger-rich cysteine repeat (SRCR) domain of SAG/gp340 that is targeted by AgI/II proteins (Bikker et al., 2002, 2004). It was shown that immobilized SRCR underwent a conformational change in the presence of calcium that allowed for AgI/II protein interaction with nanomolar affinity, whereas AgI/II protein interacted with fluid-phase SRCR with micromolar affinity (Purushotham and Deivanayagam, 2014). Additionally, SAG/gp340 size variants exist that have altered interactions with AgI/II proteins from different species (Jonasson et al., 2007). Furthermore, alternate AgI/II V-domain segments within a group of *S. mutans* human isolates were shown to confer differential saliva- and SAG/gp340-mediated adherence (Esberg et al., 2012, 2017). Taken together, this information suggests a complex co-evolution of AgI/II proteins and SAG/gp340. While fluid-phase SAG/gp340 normally functions as part of host innate immunity to cause aggregation and clearance of oral streptococci, certain species have evolved to interact with the alternative conformation of immobilized SAG/gp340 on tooth surfaces to promote adherence instead. This provides a mechanism by which both commensal and cariogenic streptococci maintain colonization of the oral cavity. The role of interactions between AgI/II proteins with SAG/gp340 in other diseases will be discussed further on.

Streptococcal AgI/II proteins are able to interact with other microbes known to colonize the oral cavity, contributing to polymicrobial biofilm formation. Often, these interactions stabilize the colonization of pathogens associated with the development of periodontitis or dental caries. While SAG/gp340 has been shown to increase adherence of both *S. mutans* and *S. sobrinus* to immobilized early plaque formers such as *S. sanguinis* and *Actinomyces viscosus*, both species were also shown to adhere to the plaque formers in an AgI/II dependent manner, even without the addition of saliva or purified SAG/gp340 (Lamont et al., 1991). *S. gordonii* SspA and SspB have similarly been shown to interact with *Actinomyces oris* (Back et al., 2015) and the periodontitis-associated pathogen *Porphyromonas gingivalis* (Brooks et al., 1997; Daep et al., 2006). In the case of *P. gingivalis*, it was determined that the minor fimbrial antigen (Mfa1) of *P. gingivalis* interacts with a specific sequence motif found in the C-terminal domain of the SspA/B proteins which is not highly conserved in SpaP (Demuth et al., 2001; Daep et al., 2006; Forsgren et al., 2010). The crystal structure of SspB revealed that this region protrudes from the protein, creating a handle for initial attachment of *P. gingivalis* and inclusion into a biofilm (Forsgren et al., 2010). Peptides mimicking this region have been shown to block the SspB-Mfa1 interaction and inhibit *P. gingivalis* virulence within a murine model for periodontitis (Daep et al., 2011). Furthermore, small molecule inhibitors were recently identified that similarly block the SspB-Mfa1 interaction and attenuate the ability of *P. gingivalis* to cause periodontitis *in vivo* (Roky et al., 2020). *S. gordonii* SspA and SspB, *S. intermedius* Pas, and *S. mutans* SpaP have also all been shown to contribute to aggregation with *Actinomyces naeslundii*, another organism associated with periodontitis (Jakubovics et al., 2005b). Additionally, *S. mutans* SpaP mediates interactions with *Fusobacterium nucleatum* ssp. *polymorphum*, which in turn is known to initiate adherence of other periodontal pathogens (Guo et al., 2017).

AgI/II proteins also contribute to interactions with pathogens associated with dental caries. For example, *Lactobacillus casei* is frequently isolated from dental caries. Co-culturing *L. casei* with *S. mutans* increases the ability of *L. casei* to form biofilms, and this increase was shown to be SpaP dependent (Wen et al., 2010, 2017). Additionally, the fungal pathogen *Candida albicans* is commonly associated with oral streptococcal biofilms, and its presence has recently been shown to be positively correlated with cariogenic traits of *S. mutans* in caries-related biofilms (Falsetta et al., 2014; Bachtiar and Bachtiar, 2018). AgI/II proteins are known to interact with the Agglutinin-like sequence family proteins Als1 and Als3 of *C. albicans*, contributing to the formation of polymicrobial communities. For example, *S. gordonii* SspB interactions with *C. albicans* Als3 have been shown to contribute to biofilm formation (Bamford et al., 2009; Silverman et al., 2010). *S. mutans* SpaP was also shown to be important for incorporation of *C. albicans* within a two-species biofilm, as well as for acid production within the biofilm, which may contribute to the progression of dental caries (Yang et al., 2018).

Despite the body of work indicating the role of AgI/II proteins in polymicrobial biofilm formation with oral pathogens, it has been shown that *S. mutans spaP* is significantly downregulated within two-species biofilms with *S. sanguinis, S. oralis* or *L. casei* (Wen et al., 2010). Very little is known regarding the regulation of AgI/II family proteins, but this indicates that at least within the context of biofilm formation, the regulation of AgI/II proteins is complex and warrants further investigation. Additionally, while it is known that AgI/II proteins contribute to oral disease through adherence or even invasion of dentinal tubules (Love et al., 1997), the role of AgI/II proteins in the immune response within the oral cavity remains largely unexplored.

### Respiratory Tract

Streptococci are often colonizers of the nasopharyngeal tract and are a common cause of lung infections. The role of AgI/II proteins in streptococcal persistence within these two niches has been characterized mainly with *S. suis*, *S. gordonii*, and GAS. There is serotype-specific variation in the *S. suis* AgI/II contribution to respiratory infection. The *S. suis* AgI/II protein contributes to adherence to NPTr porcine tracheal cells in a serotype 9 strain, but not in a serotype 2 strain (Chuzeville et al., 2017). A mutant lacking the AgI/II protein in the serotype 9 strain also displayed decreased nasal cavity and tonsil bacterial burden within a porcine model for respiratory infection (Chuzeville et al., 2017). As *S. suis* is also able to cause human infections, it would be of interest to examine this in models for human disease. Studies with *S. gordonii* have used human cell-based models for disease. Some of these were done using HEp-2 cells which were initially thought to be derived from a human laryngeal epithelial carcinoma, however, it has been shown that the cell line was actually the result of HeLa cell contamination (Gartler, 1968). In order to provide historical context, these results will still be mentioned. *S. gordonii* SspA and SspB contribute to adherence and invasion of the HEp-2 cell line (Jakubovics et al., 2005a; Nobbs et al., 2007; Andrian et al., 2012). As mentioned earlier, this was shown to be mediated by recognition of β1 integrin by SspA and SspB (Nobbs et al., 2007). SspA and SspB contribute to *S. gordonii* adherence to a lung epithelial cell line (A549) through β1 integrin as well (Andrian et al., 2012). Additionally, AspA contributes to GAS colonization of the nasopharynx, as seen by its role in adherence to Detroit 562 laryngeal epithelial cells (Franklin et al., 2013). An AspA deficient mutant displayed decreased nasopharyngeal and lung bacterial burden within a murine model of respiratory infection (Franklin et al., 2013). While AspA has also been linked to innate immunity through repression of phagocytic killing (as discussed previously), continued investigation is required to determine its role in immune modulation specifically within the respiratory tract. Additionally, while β1 integrin was shown to be a receptor for SspA and SspB in HEp-2 cells, host respiratory epithelial cell receptors for AgI/II proteins have not been thoroughly characterized; however, since SAG/gp340 is known to be expressed in the lungs, this would be one logical interaction to examine.

### Female Reproductive Tract Interactions

Numerous streptococcal species are known to colonize the female reproductive tract. As seen with GBS, this can result in pregnancy-associated complications such as pre-term births. AgI/II proteins are beginning to be implicated in streptococcal colonization of these tissues as well. Recently, the role of the GBS Bsp proteins in colonization of the female reproductive tract has been described. Deletion of BspC from GBS strain 515 resulted in a modest decrease in adherence to vaginal epithelial cell (VECs); however, pretreatment with Bsp antisera resulted in a large decrease in adherence as compared to the preimmune sera control treatment (Pidwill et al., 2018). Additionally, heterologous expression of both BspA and BspC within *Lactococcus lactis* was shown to confer increased adherence to a VEC monolayer (Rego et al., 2016; Pidwill et al., 2018).

The GBS AgI/II proteins are also known to contribute to single species biofilm formation (Chuzeville et al., 2015), but there is evidence that these proteins mediate polymicrobial interactions as well. As mentioned earlier, AgI/II proteins are able to interact with the fungal pathogen *C. albicans*, which is also often co-isolated with GBS from the vaginal tract (Monif and Carson, 1998; Bayó et al., 2002; Cools et al., 2016). BspA and BspC were shown to mediate direct interaction between GBS and *C. albicans* (Rego et al., 2016; Pidwill et al., 2018). Furthermore, adherence of both GBS and *L. lactis* surrogate expression strains to VECs in the presence of *C. albicans* was largely mediated by expression of BspA and BspC. The association between GBS and *C. albicans* is dependent on the Als3 protein of *C. albicans* (Pidwill et al., 2018). Interestingly, Als3 is the same protein that was discussed earlier as being the receptor for AgI/II proteins of oral streptococci, indicating that these interactions are not niche specific. These experiments were all conducted *in vitro.* As such, the role of AgI/II interactions with *C. albicans* within the female reproductive tract *in vivo* remains unexplored.

Other reports of AgI/II interactions within the female reproductive tract are not as direct or as thoroughly studied as the Bsp interaction with vaginal epithelium, as is seen with GAS. Colonization of the genitourinary tract by GAS is a risk factor for puerperal sepsis, and it is possible that the GAS AgI/II protein contributes to colonization of this niche. GAS adherence to vaginal epithelium and colonization of murine vaginal tract was shown to be mediated by genes located on RD2 (Jain et al., 2019). As mentioned earlier, GAS RD2 contains the AgI/II protein AspA. The individual contribution of AspA to vaginal colonization, however, has not yet been shown. Future studies should additionally aim to determine what, if any, impact AgI/II proteins have on streptococcal ascending infection or immune modulation resulting in adverse pregnancy outcomes.

### Meningitis

Several streptococcal species are able to penetrate the blood-brain barrier (BBB) in order to cause meningitis, including *S. pneumoniae*, *S. suis* and GBS (Doran et al., 2016). Only in GBS has the role of AgI/II been investigated. A recent study revealed that BspC promoted GBS adherence to hCMECs, a model of the BBB, by interacting with the C-terminus of the intermediate filament protein known as vimentin (Deng et al., 2019). BspC itself induced meningitis-associated inflammation, as discussed in the immune signaling section, and the BspC-vimentin interaction was also necessary for disease progression. A BspC-deficient mutant was unable to cause meningitis within a murine model, while vimentin-deficient mice were protected from infection by wildtype GBS. Furthermore, vimentin was shown to localize to the cell surface in response to interaction with BspC, although the mechanisms governing this reorganization are not yet understood. While *S. suis* AgI/II contributions to systemic infections (discussed within the “Interactions With the Immune System” section) have been previously investigated, their role specifically within central nervous system infections should be examined in future studies.

### Infective Endocarditis

Another major example of AgI/II proteins interacting with host endothelial cells is during infective endocarditis (IE), which is characterized by the formation of a bacteria-containing vegetation on a heart valve (Ford and Douglas, 1997). Viridans group streptococci are the second largest cause of IE (Rajani and Klein, 2020) and as such, the role of AgI/II proteins in promoting IE has been widely investigated. The characteristic vegetation in IE is typically composed of fibronectin and fibrinogen, bacteria, and aggregated platelets (Baddour, 1994; Ford and Douglas, 1997). As mentioned previously, many AgI/II proteins are known to interact with both fibrinogen and fibronectin. In addition, *S. mutans* PAc binds platelets and causes their aggregation (Matsumoto-Nakano et al., 2009). *S. gordonii* SspA and SspB have also been shown to bind human erythrocytes (Demuth et al., 1996) and contribute to platelet aggregation, although they do not directly mediate platelet adhesion (Kerrigan et al., 2007).

The role of AgI/II proteins in interacting with endothelial cells is also relevant here, although many of these studies have used cell lines originating from regions distal to the heart. Interactions with brain endothelium were described in the previous section, but SpaP has also been shown to contribute to *S. mutans* adherence to the human saphenous vein endothelial cell (HSVEC) line (Vernier et al., 1996; Al-Okla et al., 1999). One study used human aortic endothelial cells (HAECs) to model *S. mutans* interactions during IE, and while they did not directly investigate the role of AgI/II proteins, they instead looked at expression of known AgI/II protein receptor DMBT1 (SAG/gp340) in response to infection (Oho and Nagata, 2019). The study found that SAG/gp340 release was upregulated in response to *S. mutans* infection, but this actually decreased adherence and invasion of the HAECs as compared to a SAG/gp340 knockdown cell line. In this case, it seems that the host utilizes the protein in a similar manner to fluid-phase SAG/gp340 to aggregate and promote clearance of *S. mutans*.

Interestingly, despite the role of AgI/II proteins in interacting with platelets, erythrocytes, and endothelial cells, as well as elevated levels of anti-PAc antibody titers in IE patients (Russell et al., 1992), the only study examining AgI/II proteins in an *in vivo* model of IE found no phenotype for an AgI/II-deficient mutant (Ryd et al., 1996). The study found no statistically significant difference in CFUs present in heart valve vegetations between the mutant and wildtype *S. mutans* strains 1-hour post inoculation, although there was a trend toward fewer CFU of the mutant strain. While they observed no difference in bacterial clearance from the blood, CFU counts were only examined for 1 hour post infection, which may have been too early in disease progression. Nonetheless, at 48 hours post infection, they still observed no difference between the wildtype and AgI/II-deficient mutant in CFU counts recovered from heart valve vegetations. Despite these results, due to the numerous differences between AgI/II protein interactions from different species that have been described thus far, revisiting an *in vivo* model for IE to investigate the contribution of AgI/II proteins from other species may provide novel insights into the development of the disease. Additionally, it is also possible that AgI/II proteins mediate IE disease through an altered immune response instead of through direct adherence. A recent study showed that there is a high level of peptide similarity between the *S. mutans* AgI/II protein and cardiovascular autoantigens (Lucchese, 2017). The role of the elevated anti-AgI/II antibody titers (Russell et al., 1992) in recognizing these autoantigens to promote IE should be investigated.

## AGI/II Proteins as Therapeutic Targets

Given the growing recognition of their role in promoting streptococcal colonization and pathogenesis, the potential for AgI/II proteins to serve as therapeutic targets warrants consideration. Reflecting the timeline for discovery of this protein family, such investigations to date have primarily focused on exploiting the AgI/II protein of *S. mutans* to develop an anti-caries vaccine. Since it was first shown that antibodies against SpaP can impair adherence of *S. mutans* to SAG immobilized on the tooth surface (Hajishengallis et al., 1992), numerous vaccine strategies have been explored, with the ultimate goal of inducing a mucosal immune response that inhibits *S. mutans* colonization of the oral cavity. Approaches include immunization with recombinant SpaP peptides or chimeric proteins incorporating additional *S. mutans* antigens (Lehner et al., 1981; Katz et al., 1993; Yu et al., 1997; Hajishengallis et al., 1998; Zhang et al., 2002; Batista et al., 2017), attenuated *Salmonella enterica* serovar Typhimurium expressing a SpaP epitope (Huang et al., 2001; Jiang et al., 2017), or DNA vaccines (Guo et al., 2004; Jia et al., 2004, 2020; Shi et al., 2012), alongside passive immunotherapy studies using monoclonal antibodies (Lehner et al., 1985, 1992). While most of these studies have been at the preclinical phase using animal models, human studies involving topical application to the tooth surface of anti-SpaP monoclonal antibodies were shown to be effective in preventing colonization by exogenously administered or indigenous *S. mutans* (Ma et al., 1989, 1998). Protection was reported to last for up to 2 years (Ma and Lehner, 1990; Ma et al., 1990), although others failed to replicate such long-term effects (Weintraub et al., 2005), and no follow-up trials have been performed in recent years. Alongside the technical difficulties of vaccine development, another challenge with an anti-caries vaccine is that *S. mutans* is not the only cariogenic bacterium within the oral microbiota, which raises the question of the efficacy of targeting a single bacterium. However, this issue is less of a concern for the development of a vaccine to combat, for example, GBS disease. Sialylated capsular polysaccharide (CPS) has been a major focus of GBS vaccine strategies to date (Kobayashi et al., 2016). However, there are 10 distinct CPS serotypes, as well as non-typeable strains, meaning that any such vaccine design could face the challenge of potential serotype replacement/switching across geographical sites and over time. Vaccines comprising a combination of protein-based antigens have potential to confer broad protection that is serotype-independent, and these are currently under investigation. Based on SpaP, the Bsp proteins or domains thereof might well be anticipated to induce function-blocking antibodies, while their unique structural features offer the potential for the identification of epitopes that allow the selective targeting of GBS while not impacting other streptococci within the resident microbiota at a given ecological niche.

Alongside vaccination strategies, improved knowledge of structure-function relationships across the AgI/II proteins offers the potential for mimetics or small molecule inhibitors to be developed as a therapeutic route. As mentioned, proof-of-concept has been established with use of a peptide to block the interaction of *S. gordonii* AgI/II with *P. gingivalis* Mfa1, resulting in reduced alveolar bone resorption in a murine model of periodontitis (Daep et al., 2011). More recently, this peptide has been incorporated into polymeric nanoparticles to improve peptide dosage and persistence within the oral cavity (Mahmoud et al., 2018), while small molecule inhibitors of the AgI/II-MfaI engagement have also been found to effectively reduce *P. gingivalis* virulence (Roky et al., 2020). Protective effects have also been shown for a peptide (p1025) that blocks *S. mutans* AgI/II adhesion to SAG. In a human trial, topical application of p1025 to the teeth over a 3-week period prevented recolonization by *S. mutans* for up to 4 months but not by another member of the resident oral microbiota, *Actinomyces* (Kelly et al., 1999). With our growing understanding of the importance of the AgI/II protein family amongst pathogenic streptococci, opportunities for development of therapeutic agents that target them should remain an important area of investigation.

## Conclusion and Future Directions

**FIGURE 3:**
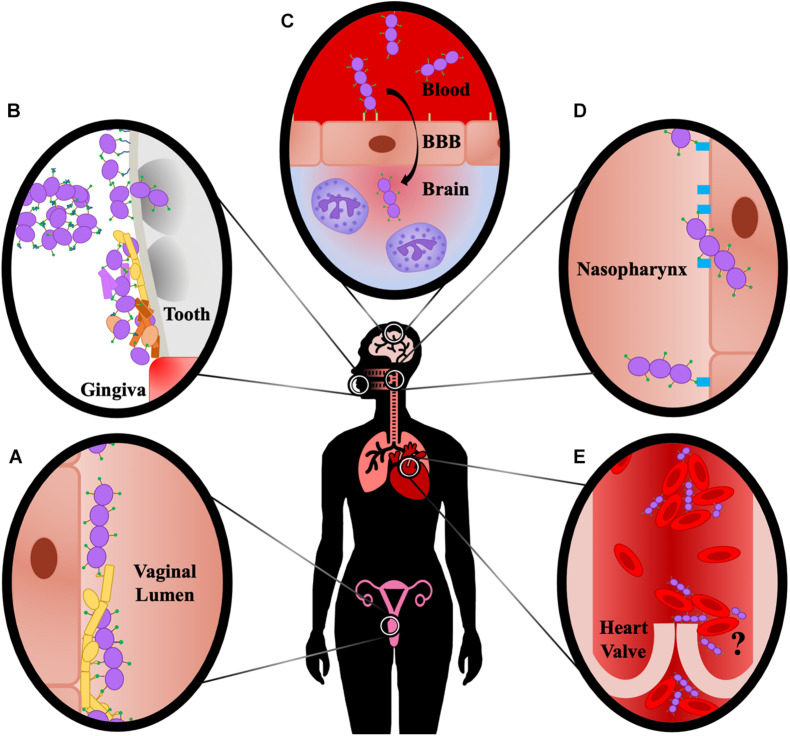
Summary of Site-Specific AgI/II Interactions. **(A)** AgI/II proteins promote direct interactions with vaginal epithelial cells, and interact with Als3 protein of *C. albicans*, promoting fungal and streptococcal adherence to the vaginal epithelium. **(B)** AgI/II proteins bind fluid-phase SAG/gp340, resulting in aggregation and clearance through mechanisms such as swallowing, as well as to immobilized SAG/gp340 on the tooth pellicle, facilitating adherence and colonization. AgI/II proteins mediate direct adherence to tooth components and invasion into dentinal tubules, resulting in caries formation, and also facilitate colonization and polymicrobial biofilm formation with cariogenic pathogens, as well as with pathogens associated with periodontitis. **(C)** AgI/II proteins interact with vimentin on the surface of brain endothelium, and also induce chemokine signaling and neutrophil chemotaxis characteristic of meningitis. **(D)** AgI/II proteins interact with β1 integrin on the nasopharynx epithelium, resulting in adherence and invasion of the cells and consequently increased bacterial burden in the nasopharynx and lungs. **(E)** AgI/II proteins promote aggregation of blood-borne bacteria, as well as attachment to and aggregation of platelets; however, there is currently insufficient evidence to show this promotes increased vegetation formation on heart valves that is characteristic of infective endocarditis, as indicated by the question mark.

The AgI/II family of proteins display both overlapping and diverse functions that vary between streptococcal species as well as the niche within the host (Figure 3). The protein structure itself has many unique features, with multiple intramolecular interactions forming an elongated fibrillar stalk that projects the V-domain containing a binding cleft away from the cell surface. This cleft may be used to promote streptococcal colonization of various tissues via both direct cellular adherence or interaction with ECM components. Here we provide a historical perspective of the existing literature and as such, the data presented represent over 40 years of work. During this time, the field has progressed significantly. Some of the pitfalls in the earlier studies that have become clear in hindsight were discussed. In general, validated cell lines, antibodies, recombinant proteins made with the benefit of current structural knowledge, and other modern tools and techniques could provide clarification or new insights into some of the previously identified functions of AgI/II proteins. In addition to the roles for AgI/II proteins in mucosal colonization and blood-borne diseases that were discussed, there are also functions that have yet to be investigated in detail. For example, SspA and SspB protect *S. gordonii* from the antibiotic polymyxin B, as well as the antibacterial peptides nisin and histatin-5 (Andrian et al., 2012). AgI/II proteins may also play a role in streptococcal gut colonization, as the *S. suis* AgI/II was shown to protect against acid stress (Chuzeville et al., 2017), and a potential AgI/II protein identified in *S. salivarius* contributed to adherence to intestinal epithelium (Chaffanel et al., 2018). Each of these potential functions should be further investigated. Additionally, future studies should aim to elucidate the potential for post-translational modifications of AgI/II proteins such as glycosylation to contribute to their functions. Understanding the diverse nature of these multifunctional adhesins will provide novel insights into the mechanisms by which streptococci and other organisms with structurally similar adhesins cause disease, as well as provide targets for therapeutic intervention.

## Author Contributions

HM, AN, and KD contributed to writing, reviewing, and editing the manuscript. HM generated the table and figures. All authors contributed to the article and approved the submitted version.

## Conflict of Interest

The authors declare that the research was conducted in the absence of any commercial or financial relationships that could be construed as a potential conflict of interest.
